# Bone Invasive Meningioma: Recent Advances and Therapeutic Perspectives

**DOI:** 10.3389/fonc.2022.895374

**Published:** 2022-06-30

**Authors:** Hajime Takase, Tetsuya Yamamoto

**Affiliations:** ^1^ Center for Novel and Exploratory Clinical Trials (Y-NEXT), Yokohama City University Hospital, Yokohama, Japan; ^2^ Department of Neurosurgery, Graduate School of Medicine, Yokohama City University, Yokohama, Japan

**Keywords:** meningioma, bone invasion, recurrence, translational study, long-term

## Abstract

Meningioma is the most common primary neoplasm of the central nervous system (CNS). Generally, these tumors are benign and have a good prognosis. However, treatment can be challenging in cases with aggressive variants and poor prognoses. Among various prognostic factors that have been clinically investigated, bone invasion remains controversial owing to a limited number of assessments. Recent study reported that bone invasion was not associated with WHO grades, progression, or recurrence. Whereas, patients with longer-recurrence tended to have a higher incidence of bone invasion. Furthermore, bone invasion may be a primary preoperative predictor of the extent of surgical resection. Increasing such evidence highlights the potential of translational studies to understand bone invasion as a prognostic factor of meningiomas. Therefore, this mini-review summarizes recent advances in pathophysiology and diagnostic modalities and discusses future research directions and therapeutic strategies for meningiomas with bone invasion.

## Introduction

### Meningiomas

Meningioma is the most common primary neoplasm of the central nervous system (CNS) in adults, originating from arachnoid cap cells covering the CNS. They are classified according to histopathological characteristics and have a broad morphological spectrum, reflected in 15 subtypes. (1–3). The World Health Organization (WHO) has also classified meningiomas into three grades (1–3), similar to other CNS tumors, linked to overall expected clinical-biological behaviors. Most tumors are WHO grade 1, which are slow-growing with benign features and a comparatively good prognosis. WHO grades 2 and 3 (4, 5) have local brain invasiveness and cellular features, including higher mitosis and atypia. In general, symptomatic cases of any WHO grade are surgically treated, and to date, there is no consensus on the effectiveness of pharmacotherapy, including chemotherapy (6). Hence, Simpson’s grade, based on the extent of surgical resection, has been considered a good tumor recurrence indicator in addition to WHO grading (7). Simpson grade I is defined as complete removal, including resection of the underlying bone and associated dura. However, meningiomas classified as WHO grade 1 and Simpson grade I sometimes recur in long-term follow-up, often requiring additional treatments, such as secondary surgery or salvage radiosurgery, which can be challenging and potentially lead to morbidity (8). Therefore, recent studies have highlighted the importance of long-term recurrence prediction with a different viewpoint than WHO grade and developing diagnostic and therapeutic options for such recurrent cases.

### Meningioma *With Bone Invasion*

Meningiomas are categorized inconsistently based on their location (9). Sometimes, meningiomas grow extracerebrally, corresponding with the tumor’s origin. Tumors arising from locations other than the subdural compartment have been termed ectopic, extracranial, extraneuraxial, extradural, or intraosseous meningiomas (9, 10).

**Figure 1:**
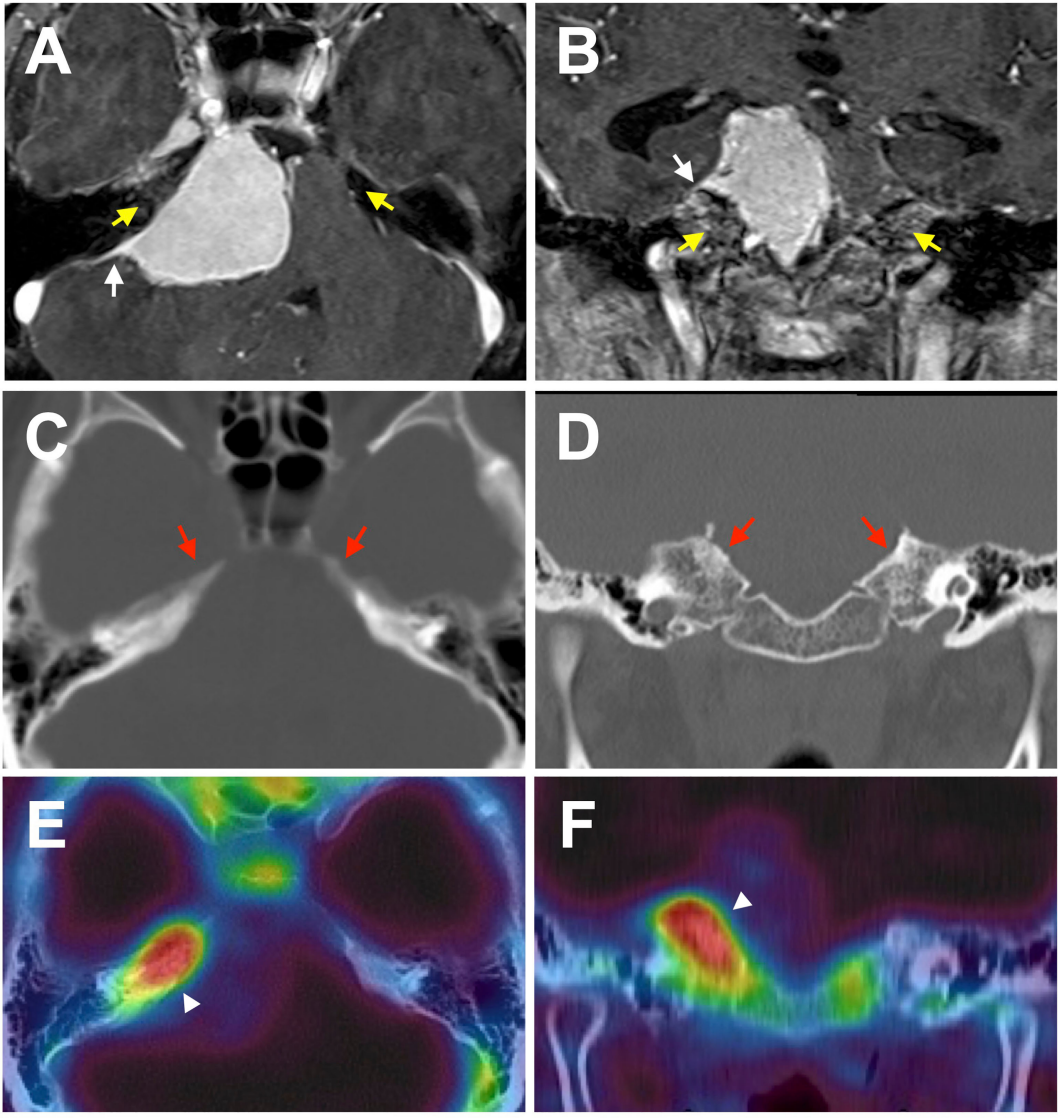
A representative case of meningioma with bone invasion. Axial **(A, C, E)** and coronal **(B, D, F)** images demonstrating a petroclival meningioma. **(A, B)** T1-post contrast MRI shows a characteristic dural tail (white arrows). Enhancement in the adjacent bone is ambiguous, and no obvious laterality is found (yellow arrows). **(C, D)** Non-contrast bone CT does not reveal a hyperostosis with tumor-associated laterality. **(E, F)** However, F^18^ fluoride PET/CT fusion image indicates prominent uptake in the adjacent bone suggesting bone invasion of the tumor (white arrowheads).

Primary intraosseous meningioma usually describes tumors that develop mainly from the calvarium and are unequivocally excluded from the subdural component (11). Contrastingly, many unrecognizable meningioma synonyms and subtypes secondarily extend into the adjacent bone, such as secondary intraosseous meningioma, meningioma with bone infiltration, and meningioma with bone invasion. (In this review, they are consistently noted as bone-invasive meningiomas to avoid confusion). In general, bone invasive meningiomas can be preoperatively diagnosed by conventional radiographic modalities, such as magnetic resonance imaging (MRI) and computed tomography (CT). They are histopathologically confirmed after surgery, since the preoperative judgment of bone involvement is sometimes ambiguous (**Figures 1A–D**). Meningioma en plaque, a relatively uncommon and unique form accounting for 2-9% of meningiomas, is often accompanied by hyperostosis in the middle fossa and sphenoid wing, with an incidence rate of 13–49% (12–14). Nevertheless, hyperostosis is seen less in other meningiomas except for lymphoplasmacyte-rich meningioma, a rare histologic subtype (WHO grade 1), which can arise as an en-plaque meningioma, and is characterized by a prominent infiltration of plasma cells and lymphocytes with a variable proportion of meningothelial elements (15–17). To date, hyperostosis has been thought as due to direct tumor invasion to adjacent bone and reactive hypervascularity of the periosteum leading to benign formation, and thus can often be classified as bone-invasive meningiomas (18–22).

In addition to histopathological aggressiveness and surgical extension, accumulating evidence suggests that bone invasion could predict recurrence and is possibly associated with reduced progression-free and overall survival, even in WHO grade 1 or 2 cases that surgically achieved total removal (Gabeau-Lacet et al.: Simpson I-III in WHO grade 1, Abdelzaher et al.: Simpson grade I-II in WHO grade 2, Lemee et al.: Simpson grade I-III in WHO grade 1-3) (23–25). However, due to the limited assessability, bone invasion as a recurrent predictor remains less understood, and is therefore not reflected in the WHO grading criteria. Taken together, these facts strongly suggest that further integrative study of bone invasive meningioma may provide deeper understanding of bone invasive meningioma and improve the long-term prognosis.

### Current Issues

The rarity of bone-invasive meningioma may contribute to the limited number of assessments. Thus, bone-invasive meningiomas has not been well described compared with primary intraosseous meningioma (9, 26–28). Another obstacle is the lack of a standard assessment method for bone-invasive meningiomas, except tissue histopathology. In other words, diagnostic options for meningiomas with bone invasion have been less studied. In addition, the specific mechanism of cellular infiltration and the molecular background characteristics are ambiguous. Overall, these facts result in the underdevelopment of therapeutic alternatives for invasion, except for direct microscopic surgery.

However, clinical evidence of bone-invasive meningioma is increasing, emphasizing the importance of further studies to understand bone invasion as an independent prognostic factor or a preoperative factor related to the extent of surgical resection. Several diagnostic modalities have been developed for meningiomas, including bone invasion. Furthermore, recent molecular biology advances exploring therapeutic targets provide future opportunities to reorganize meningioma issues (3, 29).

### Aims

Therefore, this mini-review briefly summarizes recent advances in the clinical knowledge of bone-invasive meningioma as a long-term recurrent predictor and introduces potent diagnostic options and molecular pathophysiology. Finally, we discuss future research directions and therapeutic strategies for meningiomas with bone invasion.

## Bone Invasion as a Predictor of Recurrence

In a surgical series of WHO grade 2 (atypical) meningiomas, as expected, several studies reported a significant association between bone invasion and progression, multiple recurrences, and poor outcomes, even in patients who underwent gross total resection (23, 30–32). In contrast, a surgical series of non-neurofibromatosis type 2 (*NF2*) cases (WHO grade 1; N = 118, grade 2 or 3; N = 26) reported that bone invasion, dural tails (identified by conventional MRI), and reactive hyperostosis (assessed by CT) were not associated with WHO grades, progression, or recurrence (33). Additionally, in a recent large series of WHO grade 1 studies, such as Corniola et al. (N = 1352) and Haddad et al. (N = 239), bone invasion was not associated with progression or recurrence (34, 35). However, patients with post-median recurrence (>24 months after treatment) tended to have a higher incidence of histopathological bone invasion (38.5% vs. 16.9% without recurrence, p = 0.064). Furthermore, Cox regression analysis identified an independent relationship between recurrence and incomplete (subtotal) resection, even in WHO grade 1 tumors with a consistent Simpson’s grade (35). Therefore, a long-term clinico-radiological study with histopathological assessment of bone invasion may be preferable to understand how bone invasion affects the recurrence of WHO grade 1 meningioma.

### Bone Invasion as a Preoperative Factor to Determine the Extent of Surgical Resection

As previously mentioned, the extent of surgical resection quantified by Simpson grade is the main predictor of recurrence. Microsurgery is “tailor-made” according to the size, surrounding structure, and anatomical location of the tumor, yet complete resection is rarely achieved. Therefore, preoperative factors for determining the extent of surgical resection are also important for predicting the prognosis during the early therapeutic stage (35, 36).

A recent surgical series incorporating retrospectively and prospectively collected data included 1469 meningiomas of all three WHO grades (1, 92.3%; 2, 5.2%; 3, 2.2%) and analyzed predictive factors related to the surgical extent of resection (25). In the largest study among a similar series, bone invasion (definition not addressed) was observed in 18.7% of cases and significantly associated with lower rates of a low Simpson’s grade (not defined) and gross total removal (GTR: defined as a Simpson grade I-III resection in this report) [odds ratio: 0.85 (0.73–0.99) and 0.55 (0.73–0.99), respectively]. Based on these results and the classification and regression tree recursive partitioning analysis, the authors demonstrated that the extent of resection could be very low for symptomatic cases, followed by bone invasion as the second main predictor [GTR; 79% (903/1130) of the cases without bone invasion]. Considering the surgical selection bias underlying asymptomatic cases, as the authors addressed, bone invasion would be a primary preoperative predictor of the extent of surgical resection. Furthermore, bone invasion may be an indirect predictor of meningioma recurrence (37–40).

### Bone Invasion and Clinicopathological Grading

Prior investigations have identified that aggressive imaging features are associated with clinicopathologically high-grade meningiomas and, therefore, increase the risk of progression or recurrence (41–45). To date, increasing findings remind us that bone invasion is a unique characteristic, partly resembling a high-grade phenotype, despite not being included in any WHO grading criteria of meningioma (5). As previously described, the incidence of histopathologically confirmed bone invasion in WHO grade 1 tended to be higher in the subgroup of post-median recurrence (>24 months; 38.5%) than in those with early recurrence (<24 months; 16.9%) (35). Nevertheless, another study of 304 cases (grade 1, N = 227; 2, N = 77; 3, N = 5) demonstrated a negative association between histopathological bone invasion and the WHO grade (46). These results suggest that long-term tumor recurrence-related bone invasion may be slower than grade 2 or 3 due to different mechanisms from ordinal histopathological aggressiveness, such as mitosis (**Figure 3**) (4, 20, 47). Since the meningioma characteristics vary tremendously and provide confusing results that are difficult to adopt into clinical practice, molecular biology research of bone-invasive meningioma may help identify therapeutic targets and understand the clinicopathological background, for instance, related to slower recurrence (33).

The findings detailed above highlight two emerging issues: 1) accurately providing a preoperative diagnosis of meningioma with bone invasion, especially for WHO grade 1 and 2) treating these patients without long-term morbidity. Additionally, an ongoing issue is whether meningiomas, including high-grade and/or bone invasive cases, benefit from early irradiation. Biological and diagnostic updates may be helpful in the future to clarify these issues (32, 47, 48).

## Biology and Diagnosis

### Radiological and Histopathological Diagnosis

There is no doubt that a suspected case of WHO grade 1 meningioma identified by MRI should be diagnosed and followed up. In addition, histopathological classification generally helps facilitate a clinico-biological diagnosis, although it is not mandatory in all cases (4). However, in meningiomas with bone invasion, the judgment of bone involvement is sometimes ambiguous as it is difficult to preoperatively diagnose whether the tumor has invaded the adjacent bone using conventional radiographic modalities, such as CT and MRI (**Figures 1A–D**). Nevertheless, progress has been made in several areas of meningioma diagnoses (48). Previously, bone invasion was only postoperatively detected by histopathology in suspected cases of bone resection.

**Figure 2:**
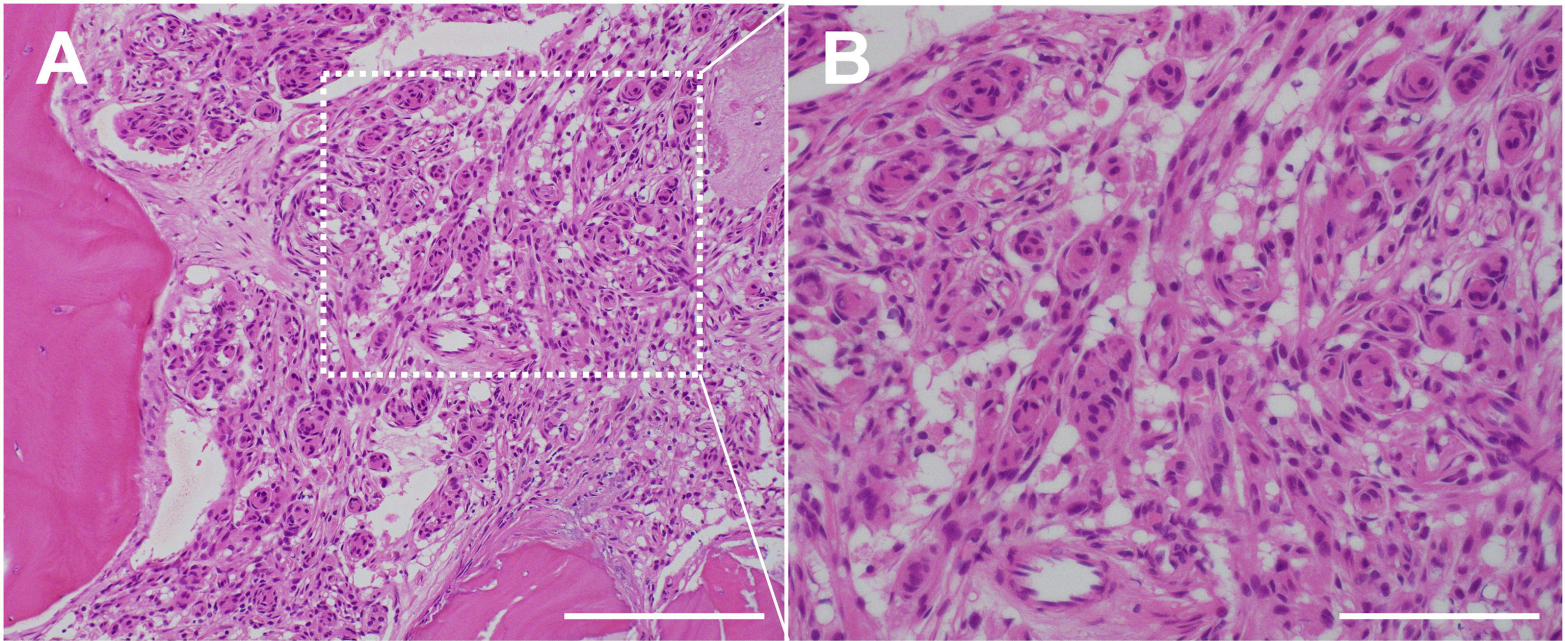
Histopathology of the case of bone invasive meningioma shown in **Figure 1**. **(A)** H&E staining demonstrating a cellular/tissue invasion into bone trabecula. ×200 magnification. Scale bar = 200 μm. **(B)** H&E staining demonstrating a proliferation of tumor cells with round to oval nuclei. Whorl formation of the tumor cells suggests meningothelial meningioma (WHO grade 1). ×400 magnification, Scale bar = 100 μm.

Hyperostosis of the bone adjacent to the meningioma, observable on CT with a bone window, has been well-described, with many reports addressing the possible causes. A primary theory is that cellular/tissue invasion of bone indicates hyperostosis (19, 49). Specifically, histopathological studies have clearly shown invasion of the tumor tissue to adjacent bone in areas of characteristic hyperostosis, possibly associated with strong somatostatin receptor subtype 2A (SSR2A) reactivity (12, 18, 50, 51). Moreover, a photodynamic diagnosis combined with histological study demonstrated the reactive fluorescence signal from the dipole to the inner table at the stump of the cranial window along with dense tumor-cells (52). Then, meningioma tissue invades lamellar bone trabeculae (53) (**Figure 2**). However, some false-negative and -positive hyperostosis cases have been diagnosed using conventional radiography (19, 51, 54). Thus, more accurate diagnostic modalities are required for meningiomas with bone invasion.

The aforementioned facts strongly suggest that the pathogenesis and molecular mechanisms underlying the cellular/tissue invasion of bone are poorly understood. This is potentially due to the high molecular and genetic heterogeneity of meningioma. Further studies for the microenvironment including bony tropism, osteolytic activity, and vascular remodeling between meningioma and the adjacent bone, that is “meningioma-bone niche”, may help in deeper understanding and future development of molecular-based therapies (55) (related to “*protein expression*” section) (**Supplementary Figure 1**).

There is growing evidence that molecular or metabolic imaging using scintigraphy or positron emission tomography (PET) is suitable for meningioma detection. Regarding bone-invasive meningioma, Gay et al. detected SSR2 *via* pre- and postoperative scintigraphy with a radiolabeled somatostatin analog ([^111^In-DTPA] octreotide) and intraoperative radio detection using a handheld gamma probe in 18 cases of meningioma en plaque. They reported that SSR2 radiodetection might help guide the surgical removal of bone invasive meningioma en plaque, pre- and postoperative management, and follow-up of meningioma with bone invasion that MRI failed to detect (56). Another study reported five cases of meningioma en plaque without previous bone decalcification, showing that all cases histopathologically were strongly positive for SSR2 and associated with intralesional features similar to oncogenic osteomalacia (51). These findings suggest a considerable limitation in the conventional radiographic assessment of meningioma with bone invasion, particularly when postoperative images are difficult to interpret and other biological and clinical implications may be provided, possibly linked to *SSR2A* expression. PET, recently developed using some somatostatin analogs, may also help detect bone invasion in meningioma (57, 58).

Whole-body ^18^F fluoride PET/CT has primarily been used in the context of possible bone metastases. Interestingly, some authors have incidentally found intense intracranial focal radiotracer accumulation in intracranial meningiomas in patients with a history of cancer (59–61). It has been suggested that ^18^F fluoride PET/CT may allow for the detection of bone invasion in meningiomas (**Figures 1E, F** **;** **Figure 2**) (62, 63). Nevertheless, the accumulation of ^18^F fluoride theoretically indicates pathological bone diseases that affect osteoblast activity, osteoclast-osteoblast interaction, and bone perfusion. Therefore, ^18^F fluoride PET can detect various metabolic, autoimmune, and osteogenic bone disorders (64). However, it is necessary to remember that ^18^F fluoride PET may provide false-positive lesions rather than bone invasion of meningiomas.

Radiomics is a novel imaging technique in the medical field, providing data regarding the biological properties and heterogeneity of the tumor by extracting many high-throughput imaging features (65, 66). Recently, radiomics has presented the possibility of accurately predicting meningioma grades and histological subtypes (67). Furthermore, preoperative imaging has the potential for predicting meningioma bone invasion (68). Zhang et al. evaluated 490 meningioma cases, of which 213 were bone-invasive meningioma primarily defined by surgeons (WHO grade 1; N = 448, 2; N = 38, 3; N = 4; the subtypes were not reported). They reported that radiomics contributed to the amelioration of clinical decision-making and bone invasion meningioma predictions, indicating that future radiomics studies with histopathologically diagnosed cases may be worthwhile to determine the value of radiomics for preoperatively diagnosing bone invasion meningioma (68, 69).

### Cytogenesis and Genomics

As previously mentioned, even histologically benign meningiomas may show invasive behavior in the adjacent bone, resulting in repeated recurrences. This phenomenon can occur even after complete macroscopic resection (7, 70, 71). These are some of the main reasons for accelerating cytogenetics of meningioma, which has been best studied in humans (72) and well-summarized in the literature (73). Briefly, meningiomas typically have a normal karyotype or losses, which are mostly monosomy, but on rare occasions, there are deletions of the tumor suppressor gene *NF2* located on chromosome 22 (74). Additionally, recent studies using next-generation sequencing approach have identified several mutations, such as *TRAF7*, *KLF4*, *AKT1*, *SMO*, and *PIK3CA*, with an interesting finding that mutations of these genes occur to a large degree without concurrent alteration of *NF2*, and that the clinical outcome and recurrence rate are associated with genomic subgroups (75, 76).

Most recently, technological developments have suggested that a higher rate of malignant meningiomas may be induced by increasing hypodiploidy, complex ablations, and even epigenetics (77–80). Furthermore, certain characteristics have been correlated with histological subtypes, especially copy number alterations and mutations, suggesting a greater potential for gene therapy (58, 80). Cytogenetics of lymphoplasmacyte-rich meningioma, a rare type of WHO grade 1 arising as an en plaque meningioma, is worth investigating, to develop therapeutic strategies for bone invasive meningioma.

Even in the era of genomics, epigenomics, and transcriptomics, there are currently no valuable cytogenetic and genetic recurrent predictors for meningioma, including bone-invasive meningiomas. Therefore, approaches combining histology, multi-”omics” patterns including radiomics and genomics, and radiological data may open a window to biology-based diagnostics for meningioma, perhaps leading to stratification of the recurrent risk and aggressive behavior of such tumors.

### Protein Expression

Previous studies have revealed the presence of receptors in meningioma tissues (81–87). In particular, SSR2 has been reported in meningioma tissues, thus is being considered for clinical applications based on its molecular characteristics. SSR2 is one of the most studied molecules in bone-invasive meningioma, especially for diagnostic applications. The vascular endothelial growth factor (VEGF) pathway, including VEGF and its receptors, is involved in the dynamic blood vessel structures under normal conditions and cooperates with growth, recurrence, and development of edema of meningioma through their neovascularization effect when overexpressed (88). Although nothing has been reported regarding the VEGF pathway in meningiomas with bone invasion, this angiogenic molecular system is now thought to be a therapeutic target (**Supplementary Figure 1**).

Matrix metalloproteinases (MMPs), a family of calcium-dependent zinc-containing peptidases, are assumed to promote tumor cell growth and invasion (89). To date, the functional role of MMPs in meningioma biology is complex and unclear. Previous studies focused on the role of MMP2 in meningiomas with tumor recurrence and brain invasion, and produced contradictory results (55). MMP2 expression was found to be different depending on histopathological subtypes (90). A study that used high-throughput tissue microarray on bone invasive meningiomas demonstrated that key proteins are differentially expressed, and that the anatomical location of bone invasion is a key determinant of the expression pattern of MMP2, together with osteopontin (OPN) and integrin beta-1 (ITGB1) (55, 91).

Proteomics is a widely accepted screening approach for broad protein profiles that directly analyzes proteins expressed by a cell, tissue, or tumor type. Proteomic approaches for meningioma arose in 2000s, and several methods have been used to demonstrate molecular patterns (92–101). However, few studies have reported on the proteome of benign meningiomas (102, 103). Furthermore, the proteomics of bone-invasion meningioma, first described by Wibom et al., has even fewer reports. Wibom et al. evaluated 42 WHO grade 1 meningiomas (13 fibrous, 29 meningothelial, 16 bone invasive, and 26 noninvasive) by mass spectroscopy, demonstrating that the protein expression pattern distinguishes invasiveness and histological type of meningioma. Furthermore, Mukherjee et al. compared liquid chromatography-mass spectrometry-based protein profiles between WHO grades 1 and 2, including bone invasion, and indicated possible intratumoral heterogeneity, thus requiring close follow-up (104). Nevertheless, proteomics is growing as a characterizing tool for meningiomas, and features of bone-invasion tumors have yet to be identified. In addition to gene-related “omics” studies, proteomic analysis can be useful for the molecular characterization of bone invasion meningiomas.

## Treatment

### Surgery

Longitudinal volumetric studies have determined that meningiomas grow by approximately 1 cm^3^ annually. Moreover, there is a significant risk of progression for younger patients (<60 years) and those with larger tumors at the initial diagnosis (>25 mm), tumors without calcification, and tumors at specific locations (e.g., non-skull base) (69, 105). Thus, Oya et al. suggested surgical resection for asymptomatic tumors with a worsening Simpson grade after conservative management if they grow under conservative management (106). In other words, preoperative factors are essential for determining the extent of surgical resection, and bone invasion may be a preoperative factor related to incomplete resection, in addition to tumor location in the skull-base (25, 33, 35). Taken together, in cases of suspected meningioma with bone invasion, maximal resection of the adjacent bone would be preferable (107). Although, meningioma surgery is sometimes challenging due to anatomical circumstances (e.g., venous sinus involvement, arterial or cranial nerve envelopment, and extensive involvement of the base of the skull), especially in skull base cases (108–111). To achieve maximal resection of meningiomas, including the adjacent bone, a multidisciplinary surgical strategy combined with preoperative embolization may help (112). Considering that patients with bone invasion may be comparatively older and the invasive component of the bone may not be too aggressive, the risk and benefit balance must be assessed to establish a certain case selection and future surgical strategy (113–117).

Intraoperative assistance to detect the suspected bone invasion margin can be key for “complete” resection during meningioma surgery. Growing experience has demonstrated the usefulness of fluorescence guidance using 5-aminolevulinic acid (5-ALA) in meningioma surgery, especially in cases of bone invasion, in addition to intraoperative radio detection of somatostatin analog using a handheld gamma probe (52, 118–126). However, as Scheichel et al. reported, the accumulation result has a positive predictive value of 100% and a negative predictive value of 83% of 5-ALA fluorescence in meningioma bone invasion, demonstrating that it may help to improve the extent of resection. However, further studies are necessary to investigate the rate of false-negative fluorescence and its effect on progression-free survival (PFS) (126).

Recent studies have reported proliferation and invasiveness differences between meningiomas located in the skull base and other areas. Furthermore, the genetic background may differ depending on the location, even in non-*NF2* meningiomas (127–130). Given that skull-base meningioma may be less biologically aggressive than those in other locations, extensive bony resection may be too challenging even after meningioma surgery has considerably improved, especially in skull-base cases. Thus far, it is unclear whether surgical resection plays a central role in meningioma treatment, and radiological follow-up is favorable in cases with suspected bone invasion. Therefore, patients with bone invasion may need additional treatments and future medical therapy in addition to those with WHO grades 2 and 3.

### Radiosurgery

Radiosurgery is an alternative for small to medium-sized symptomatic or recurrent meningiomas. Patients with large or post-surgical remaining tumors are also eligible for fractionated radiosurgery (4, 58, 131). To date, therapeutic strategies combining surgery and (fractionated) radiosurgery have been developed. However, details regarding its use based on the WHO grade, tumor size, and the anatomical location remain controversial (111).

Radiosurgery for the bone-invading component of meningiomas has been less studied. However, accumulating evidence highlights that adjuvant radiosurgery improves local control in WHO grade 2 meningiomas irrespective of the initial resection extent compared to observation only. Furthermore, bone invasion might be associated with multiple recurrences. (32, 132). One study evaluated a cohort with mixed WHO grades who underwent irradiation, reporting that PFS did not differ between cases with and without bone invasion. These results suggest that radiation may influence meningioma tissue invading the bone (48). However, the major problem with radiosurgery for bone invading meningioma is target delineation (133). Brastianos et al. suggested that radiation is unnecessary for the dural tail unless they contain suspicious nodular enhancement because they are typically composed of benign and hypervascular tissue. Additionally, WHO grade 1 and radiographically presumed grade 1 meningiomas require a 0–5 mm clinical target volume margin. In contrast, in cases of WHO grade 2 and 3 meningiomas, hyperostosis or direct bone invasion should be included in the gross tumor volume with an additional margin of 3–5 mm (134). Future prospective studies combining radiosurgery with reproducible target planning and image, and histopathology-based therapeutic strategies are needed to set up target delineation for bone-invasive meningiomas.

Recent progress in advanced radiation therapies has resulted in possibilities for the development of future treatments for bone-invasive meningiomas, especially high-grade meningiomas (135–145).

Proton beam therapy and photon radiation therapy are shown to be safe and effective for meningioma treatment (146–149). Intensity-modulated radiation therapy (IMRT) provides some benefits, such as higher dose conformality and improved target coverage, without the contraindications of conventional radiosurgery. It has also demonstrated preferable results for the treatment of meningioma causing visual impairment by minimizing toxicity to the adjacent nervous structure (148). Boron neutron capture therapy (BNCT) is a targeted radiotherapy that enables the selective elimination of malignant cells and the sparing of surrounding normal cells. Although evidence of BNCT for meningioma treatment is not as robust, recent studies have shown relatively good local control and favorable survival along with an acceptable safety profile for recurrent and refractory high-grade meningioma (139, 144). Photodynamic therapy (PDT) adopts a photosensitizer (PS) accumulated into tumor tissue or hypervascular lesion. Irradiation of the PS with a laser at a specific wavelength causes a photochemical reaction and produces singlet oxygen, resulting in cellular injury of the target (141). This mechanism causes an inherent selectivity of the procedure. Since the laser light can only penetrate a few millimeters of tissue, therapeutic potential of PDT is limited for the tumor located in deeper areas (150). Thus, PDT is lacking sufficient clinical evidence for meningioma treatment. However, studies suggest adequate effectiveness in the treatment of high-grade meningioma, in an *in vitro* environment (141, 142). Although the effectiveness of these modalities for bone-invasive meningioma is not well understood, appropriate applications should be studied according to modality-specific advantages and disadvantages (136, 142, 151).

These findings suggest that radiosurgery for bone invasion remains controversial but may show greater potential for prognosis, and further prospective studies are warranted.

### Medical Therapies

Compared with surgery and radiosurgery, studies on the clinical application of medical therapies against meningiomas are growing slowly, and some have promising results. However, the European Association of Neuro-Oncology recommended only experimental systemic therapy, with a “C” level class of evidence. Thus, no specific recommendations are provided (58, 73). The National Comprehensive Cancer Network recommends using alpha-interferon, somatostatin receptor agonists, and VEGF inhibitors to treat meningioma (152). However, their efficacy is limited. Thus far, there is no established evidence for their use, and more studies are required to unravel the mechanistic roles in bone-invasive meningiomas and across the entire meningioma spectrum (58, 78, 80, 153–155).

*In vitro* cell culture is widely used for oncological investigations, including meningiomas (156). This model provides self-mitogenic agents, autocrine mechanisms, and several molecules for developing novel systemic therapies for meningioma in the future. Studies have demonstrated the effects of signaling suppression on tumor invasion and cell proliferation, highlighting the importance of exploring novel non-toxic drugs for aggressive meningiomas (157–159). However, importantly, cell lines may harbor genomic and transcriptomic alterations, confounding translational research (160). Therefore, primary tumor culture should be performed rather than using transcriptionally different cell lines to understand the molecular mechanisms underlying meningioma invasion and cell proliferation for clinical applications, although there are limitations in availability and logistical concerns (160). Primary culture and specific cell lines have not been established for invasive bone meningiomas. A bone-like culture system formed with minerals structuring pores and a bone-like mechanical environment, similar to those used for other bone tumor research (161), and related assessment methodologies may help specify the molecular characteristics and further provide information on a novel concept of a meningioma-bone niche (**Supplementary Figure 1**).

## Summary and Therapeutic Perspectives

**Figure 3:**
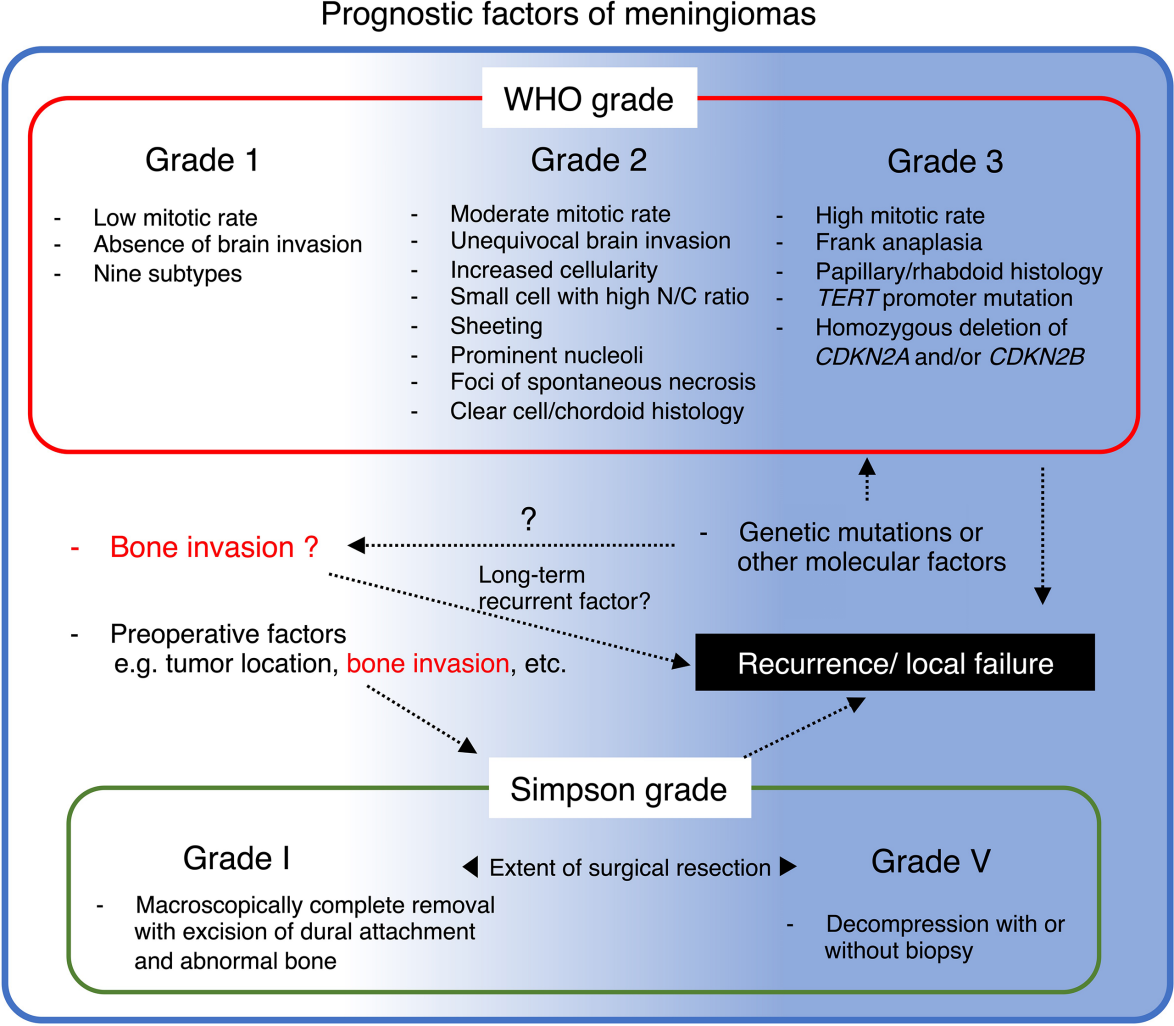
Summary of prognostic factors of meningioma and their potential relationship with bone invasion.

The abundance of clinical results and advancing research technologies have prompted the exploration of the biological characteristics of bone-invasive meningiomas. Studies have confirmed a significant association between bone invasion and incomplete resection, possibly affecting long-term recurrence and outcomes (**Figure 3**). Moreover, radio detection and fluorescence-guided 5-ALA are confirmed intraoperative assistance tools. If metabolic imaging, such as ^18^F fluoride PET, is available in addition to a precise combination of CT and MRI, suspected bone invasion can be diagnosed preoperatively. However, postoperative histopathology of adjacent bone remains a crucial part for definitive diagnosis. Advanced preoperative diagnostic modalities, such as radiomics and PET with SSR may play a central role in developing a surgical strategy for suspected cases of bone invasion.

Combined direct surgery and radiosurgery is also becoming more common, and advanced radiations, such as IMRT, BNCT, and PDT, might be good candidates for treating bone-invasive meningioma in the clinic. The specific genomic pattern of bone-invasive meningioma has not been detected. However, proteomics suggests that the protein profile of bone-invasive meningioma is more heterogeneous than that of non-invasive tumors, requiring closer follow-up. Although there is no medical therapy to treat meningiomas, including bone-invasive cases, some medical therapies are promising druggable targets, and their implementation in clinical practice is under consideration. An *in vitro* cell culture model would be a good option to test potential therapeutic targets in bone-invasive meningioma. However, primary culture should be used rather than a transcriptionally different cell line. Bone-like culture systems used for other bone tumor research may help specify the molecular characteristics and mechanisms in meningioma-bone niche, and effects of therapeutic agents for bone-invasive meningiomas.

Translating emerging clinical and basic research knowledge into clinical management remains incipient. Thus, similar to other biomedical research fields, “a valley of death exists between basic and clinical research” (162). The clinicopathological characteristics of bone-invasive meningioma are divergent, and it is challenging to commit to a long-term result when treating these tumors. However, collaborative efforts between basic science and clinics and among clinical experts, such as surgeons, radiosurgeons, radiologists, pathologists, clinician-scientists familiar with basic research, and statisticians, would help cross the valley (163).

## Author Contributions

Conception and design, drafted and/or critically revised the work, funding acquisition, Final approval of manuscript: HT; Revised the manuscript: HT and TY. All authors contributed to the article and approved the submitted version.

## Funding

This work was supported in part by Japan Society for the Promotion of Science “KAKENHI” (20K09330) (HT).

## Conflict of Interest

The authors declare that the research was conducted in the absence of any commercial or financial relationships that could be construed as a potential conflict of interest.

## Publisher’s Note

All claims expressed in this article are solely those of the authors and do not necessarily represent those of their affiliated organizations, or those of the publisher, the editors and the reviewers. Any product that may be evaluated in this article, or claim that may be made by its manufacturer, is not guaranteed or endorsed by the publisher.

## Supplementary Material

Supplementary Figure 1 “Meningioma-bone niche”, a microenvironment, in which meningioma shows bone invasion. SSR2A, strong somatostatin receptor subtype 2A; VEGFR, vascular endothelial growth factor receptor; VEGF, vascular endothelial growth factor; MMP2, matrix metalloproteinase; OPN, osteopontin; ITGB1, integrin beta-1.

## Abbreviations

CNS, central nervous system; WHO, World Health Organization; NF2, neurofibromatosis type 2; CT, computer tomography; MRI, magnetic resonance imaging; 5-ALA, 5-aminolevulinic acid; GTR, gross total removal; OR, odds ratio; PET, positron emission tomography; SSR2, somatostatin receptor subtype 2; VEGF, vascular endothelial growth factor; IMRT, intensity modulated radiation therapy; BNCT, boron neutron capture therapy; PDT, photodynamic therapy.
