# Supplementary figures and images for: Bone Invasive Meningioma: Recent Advances and Therapeutic Perspectives
Source: Front Oncol. 2022 Jun 30;12:895374. doi: 10.3389/fonc.2022.895374 (PMC9280135; doi:10.3389/fonc.2022.895374)

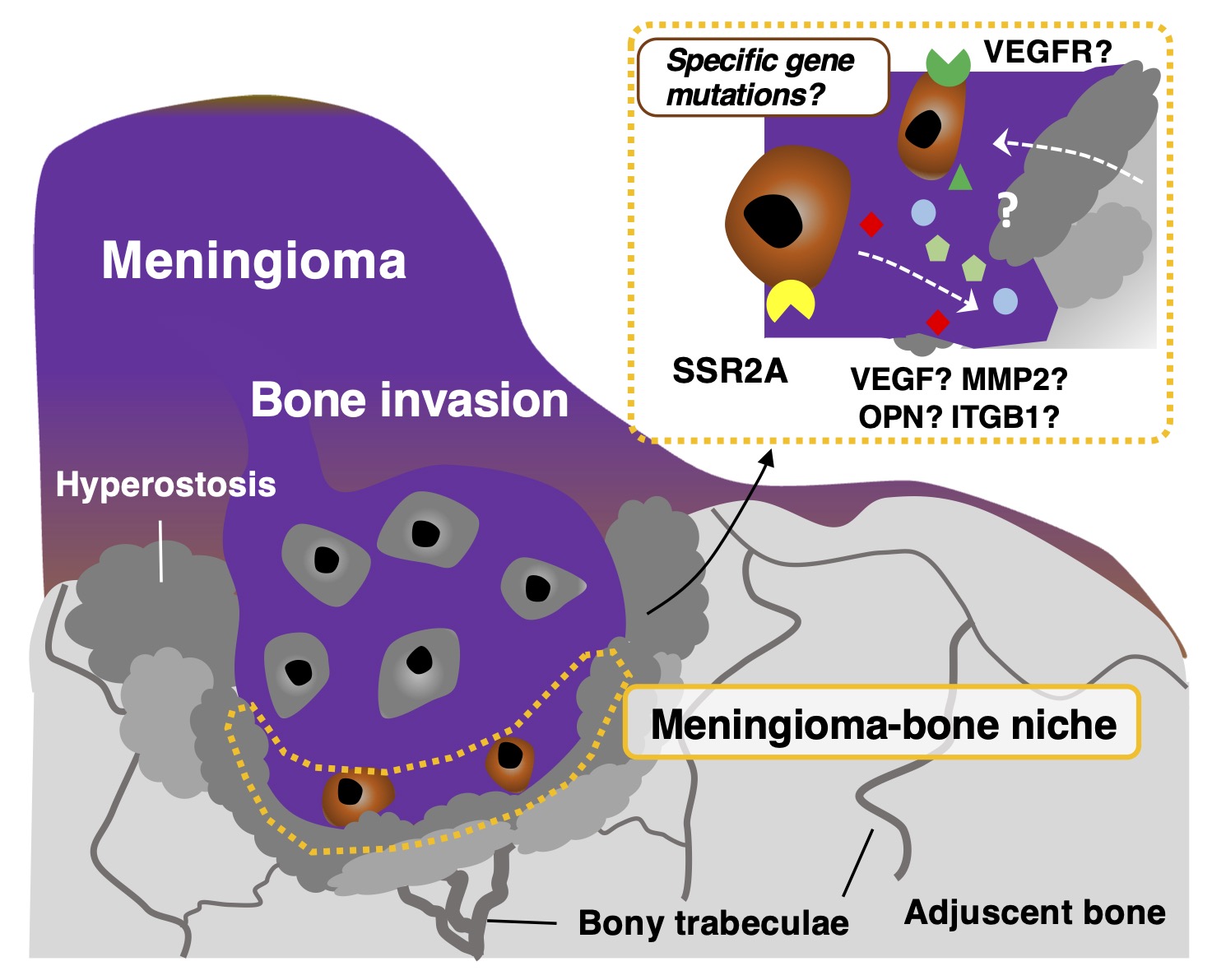

Supplement: Supplementary Figure 1 — “Meningioma-bone niche”, a microenvironment, in which meningioma shows bone invasion. SSR2A, strong somatostatin receptor subtype 2A; VEGFR, vascular endothelial growth factor receptor; VEGF, vascular endothelial growth factor; MMP2, matrix metalloproteinase; OPN, osteopontin; ITGB1, integrin beta-1. [file Image_1.jpeg]
